# DDAH1 regulates apoptosis and angiogenesis in human fetal pulmonary microvascular endothelial cells

**DOI:** 10.14814/phy2.14150

**Published:** 2019-06-17

**Authors:** Jennifer K. Trittmann, Hanadi Almazroue, Yi Jin, Leif D. Nelin

**Affiliations:** ^1^ Pulmonary Hypertension Group Center for Perinatal Research Abigail Wexner Research Institute at Nationwide Children's Hospital Columbus Ohio; ^2^ Department of Pediatrics The Ohio State University Columbus Ohio

**Keywords:** Asymmetric dimethyl L‐arginine, nitric oxide, proliferation, pulmonary hypertension

## Abstract

Nitric Oxide (NO) is an endogenous pulmonary vasodilator produced by endothelial NO synthase (eNOS). Asymmetric dimethyl L‐arginine (ADMA) is an endogenous inhibitor of eNOS activity. In endothelial cells, ADMA is hydrolyzed to L‐citrulline primarily by dimethylarginine dimethyl‐aminohydrolase‐1 (DDAH1). We tested the hypothesis that DDAH1 expression is essential for maintaining NO production in human fetal pulmonary microvascular endothelial cells (hfPMVEC), such that knockdown of DDAH1 expression will lead to decreased NO production resulting in less caspase‐3 activation and less tube formation. We found that hfPMVEC transfected with DDAH1 siRNA had lower NO production than control, with no difference in eNOS protein levels between groups. hfPMVEC transfected with DDAH1 siRNA had lower protein levels of cleaved caspase‐3 and ‐8 than control. Both DDAH1 siRNA‐ and ADMA‐treated hfPMVEC had greater numbers of viable cells than controls. Angiogenesis was assessed using tube formation assays in matrigel, and tube formation was lower after either DDAH1 siRNA transfection or ADMA treatment than controls. Addition of an NO donor restored cleaved caspase‐3 and ‐8 protein levels after DDAH1 siRNA transfection in hfPMVEC to essentially the levels seen in scramble control. Addition of a putative caspase‐3 inhibitor to DDAH1 siRNA transfected and NO‐donor treated cells led to greater numbers of viable cells and far less angiogenesis than in any other group studied. We conclude that in hfPMVEC, DDAH1 is central to the regulation of NO‐mediated caspase‐3 activation and the resultant apoptosis and angiogenesis. Our findings suggest that DDAH1 may be a potential therapeutic target in pulmonary hypertensive disorders.

## Introduction

Preterm infants are born at a time when the fetal lungs are developing, and the most preterm infants are born during the canalicular stage of lung development. Preterm birth during this vulnerable stage of lung development and the associated environmental stimuli, including supplemental oxygen and mechanical ventilation, lead to the development of the lung injury resulting in bronchopulmonary dysplasia (BPD) (McEvoy et al. 2014). Disrupted vascularization is a hallmark of BPD and a major contributor to the alveolar simplification that characterizes BPD (Mourani and Abman 2015). Furthermore, the disrupted vascularization in children with BPD puts them at very high‐risk for the development of pulmonary hypertension (PH), so called BPD‐associated PH or BPD‐PH, which is the most common co‐morbidity seen in BPD (O'Connor et al. 2016). BPD‐PH is characterized both by fewer vessels and vascular remodeling of the remaining vessels. The vascular remodeling is due to abnormal proliferation in the vessel wall and extension of vascular smooth muscle cells into normally non‐muscular arteries (Baker et al. 2014; Alvira 2016).

Nitric oxide (NO) is an endogenous pulmonary vasodilator and a key regulator of pulmonary angiogenesis (Murohara et al. 1999). NO is made by the nitric oxide synthases, of which there are three members: endothelial NOS (eNOS), neuronal NOS (nNOS), and inducible NOS (iNOS). In the pulmonary endothelium the primary source of NO is eNOS. NO is an important downstream effector of vascular endothelial growth factor (VEGF)‐mediated angiogenesis (Alvira 2016). Furthermore, eNOS deficient mice have abnormal pulmonary angiogenesis and abnormal lung development (Han and Stewart 2006). Asymmetric dimethylarginine (ADMA) is an endogenous inhibitor of eNOS and is important in the pathophysiology of endothelial dysfunction in some cardiovascular diseases in adults (Pullamsetti et al. 2005; Boger 2009; Blackwell 2010; Willeit et al. 2015). For infants with BPD‐PH, we have previously found significantly higher levels of ADMA in plasma samples from patients with BPD‐PH than in plasma samples from patients with BPD without evidence of PH (Trittmann et al. 2015). ADMA is degraded by dimethylarginine dimethylaminohydrolase (DDAH) to L‐citrulline and dimethylamine. There are two isoforms encoded by two different genes DDAH1 and DDAH2 (Leiper et al. 1999; Tran et al. 2003; Vallance and Leiper 2004; Pope et al. 2009b; Janssen et al. 2013). DDAH1 is the main isoform for ADMA degradation *in vivo* (Pope et al. 2009a; Buijs et al. 2017). Studies in transgenic mice over‐expressing DDAH1 showed lower plasma levels of ADMA, increased NO production, and NOS activity (Dayoub et al. 2003; Jacobi et al. 2005), as well as enhanced angiogenesis after ischemia or inflammation (Jacobi et al. 2005). In a variety of vascular diseases, decreased DDAH expression/activity is associated with endothelial dysfunction mediated by increased levels of ADMA causing inhibition of eNOS (Pope et al. 2009a). Endothelial DDAH1‐specific knockout mice have impaired angiogenesis (Zhang et al. 2013; Dowsett et al. 2015). DDAH1/ADMA/NOS pathway regulation of caspase‐3 mediated apoptosis has been described (Wang et al. 2016; Hou et al. 2018; Liu et al. 2018), and DDAH1 upregulation has been associated with tumor regression in a xenograft mouse model (Yung et al. 2016). Caspase‐3, in addition to its central role in apoptosis, is paradoxically known to enhance cellular growth (Laplante et al. 2010; Li et al. 2010; Kennedy et al. 2014; Feng et al. 2015), and to promote angiogenesis (Povero et al. 2013; Feng et al. 2015). We have previously found that a *DDAH1* single nucleotide polymorphism (rs480414) was protective against the development of PH in a cohort of BPD patients (Trittmann et al. 2016a). Thus, we tested the hypothesis that DDAH1 expression is essential for maintaining NO production in human fetal pulmonary microvascular endothelial cells (hfPMVEC), such that knockdown of DDAH1 expression will lead to decreased NO production resulting in less caspase‐3 activation and less endothelial cell tube formation in matrigel. We used siRNA against DDAH1 and in some experiments we utilized exogenous ADMA. We used NO donors to determine the role of NO in the DDAH1 siRNA and ADMA effects on hfPMVEC apoptosis and angiogenesis.

## Methods

### Human fetal pulmonary microvascular endothelial cells (hfPMVECs)

hfPMVECs were obtained from ScienCell Research Laboratories, Inc., Carlsbad, CA (Catalog #: 3000, Lot #: 15900, 14387, & 5016), and were used in experiments between the fourth and sixth passages. Each lot number is a cell line from a separate individual. hfPMVECs were phenotyped between passages by observation under the microscope for their typical cobblestone morphology. hfPMVECs were grown in 21% O_2_‐5% CO_2_‐balance N_2_, at 37°C in 30 mm cell culture plates according to the manufacturer's recommendations using endothelial cell medium (ECM) (ScienCell Research Laboratories, Inc., Cat #:1001).

### Transfection of DDAH1 siRNA

hfPMVECs were transfected with DDAH1 siRNA (SMARTpool: siGENOME, DDAH1 siRNA, Human, Catalog #: M‐008528‐00‐0005, Dharmacon, Lafayette, CO), or scramble siRNA as a control using Dharmafect (Dharmacon) transfection reagent as described previously (Jin et al. 2015; Nelin et al. 2016; White et al. 2017). The hfPMVECs were allowed to recover in 21% O_2_‐5% CO_2_‐balance N_2_ at 37°C for 24 h prior to experiments.

### Protein isolation

Protein was isolated from hfPMVECs, as described previously (Toby et al. 2010; Cui et al. 2011; Nelin et al. 2016). Briefly, after the experiments were completed, hfPMVECs were washed with Dulbecco's Phosphate‐Buffered Saline (DPBS) (Catalog #:0303, ScienCell Research Laboratories, Inc,) and lysis solution (20 mmol/L HEPES, pH 7.4, 50 mmol/L glycerophosphate, 2 mmol/L EGTA, 1 mmol/L DTT, 10 mmol/L NaF, 1 mmol/L Na_3_VO_4_, 1% Triton X‐100, and 10% glycerol) was added. Thirty minutes before use, the following protease inhibitors were added to each milliliter of lysis solution: 1 *μ*L aprotinin (10 mg/mL double‐distilled H_2_O), 1 *μ*L leupeptin (10 mg/mL double‐distilled H_2_O), and 1 *μ*L of phenylmethylsulfonyl fluoride (100 mmol/L/mL isopropanol). hfPMVECs were scraped and placed in sterile centrifuge tubes on ice. The samples were centrifuged at 20,000*g* for 15 min at 4°C. The supernatant was stored at −80°C for subsequent western blot analysis. Total protein concentration was determined by the Bradford method using a commercially available assay (Bio‐Rad, Hercules, CA) as described previously (Toby et al. 2010; White et al. 2017).

### Western blot analysis

Cell lysates were assayed for DDAH1, eNOS, cleaved caspase‐3, total caspase‐3, cleaved caspase‐8, total caspase‐8, cleaved caspase‐9, total caspase‐9, p21, PCNA, and *β*‐actin by western blot analysis as described previously (Nelin et al. 2005; Toby et al. 2010; Trittmann et al. 2016b; White et al. 2017). Cell lysate aliquots were diluted with SDS buffer and reducing agent, then heated to 80°C for 15 min, and centrifuged at 10,000*g* at room temperature for 2 min. Aliquots of the supernatant were used for SDS‐polyacrylamide gel electrophoresis. The proteins were transferred to polyvinylidene difluoride (PVDF) membranes and blocked overnight in Tris‐buffered saline with 0.1% Tween (TBS‐T) containing 10% nonfat dried milk. The membranes were then incubated with the primary antibody overnight. The following primary antibodies were used: DDAH1 (1:1000, Cat#: PA5‐35306, Lot#: RG2217435E, Thermo Scientific, Waltham, MA), eNOS (1:1000, Cat#:610296, Lot#:9, BD Transduction Laboratories, San Diego, CA), Cleaved caspase‐3 (1:1000, Cat#:9664, Lot#:20, Cell Signaling Danvers, MA), Total caspase‐3 (1:1000 Cat#:9662, Lot#:18, Cell Signaling), Cleaved caspase‐8 (1:1000, Cat#:9749, Lot#7, Cell Signaling), Total caspase‐8 (1:1000, Cat#: 4790, Lot#:2, Cell Signaling), Cleaved caspase‐9 (1:1000, Cat#:9505, Lot#:3, Cell Signaling), Total caspase‐9 (1:1000, Cat#:9502, Lot#:2, Cell Signaling), p21 (1:500, Cat#:471, Lot#: C196, Santa Cruz Biotechnology, Dallas, TX), and PCNA (1:5000, Cat#: P8825, Lot#: O37K4777, Sigma‐Aldrich, St. Louis, MO). The membranes were washed three times with TBS‐T. Membranes were incubated with goat anti‐rabbit IgG‐horseradish peroxidase conjugated secondary antibody (1:15,000; cat# 170‐6515, Bio‐Rad Laboratories) or goat anti‐mouse IgG‐horseradish peroxidase conjugated secondary antibody (1:10,000; cat# 172‐1011, Bio‐Rad Laboratories) for 1 h and then washed with TBS‐T. The bands of interest were visualized using Luminata Classico Western HRP substrate (EMD Millipore, Billerica, MA) and quantified by densitometry using VisionWork LS Analysis Software (UVP, Upland, CA). To control for protein loading for DDAH1, eNOS, p21, and PCNA *β*‐actin was used as described previously (Nelin et al. 2016). For cleaved caspase‐3, ‐8, and ‐9, the respective total caspase was used to control for protein loading as described previously (Trittmann et al. 2016b).

### Viable cell assay

Viable cell numbers were determined as described previously (Toby et al. 2010; Chicoine et al. 2011; Nelin et al. 2016; White et al. 2017). The same number of hfPMVECs were seeded in each well of a six‐well plate (for most experiments that number was 5 × 10^4^, for the experiments shown in Fig. 3B the number was 1 × 10^4^). In experiment shown in Figure 3A, hfPMVEC transfected with siDDAH1 or scramble were counted after trypan blue exclusion at 24, 48, and 72 h. In some experiments, ADMA 300 *μ*mol/L (Sigma‐Aldrich, St Louis, MO), 0.1* μ*mol/L DETA NONOate (Cayman Chemical, Ann Arbor, MI), 100 *μ*mol/L of the putative caspase‐3 inhibitor, Z‐DEVD‐FMK FMK004 (R&D Systems Minneapolis, MN), and/or vehicle were added to cell culture media and hfPMVECs were incubated in 21% O_2_‐5% CO_2_‐balance N_2_ at 37°C for 48 h. At the end of the experimental protocol, cells were washed twice with DPBS. After the final wash, 50 *μ*L of 0.25% trypsin diluted in 200 *μ*L of DPBS was added to each plate and incubated for 3 min, followed by the addition of 250 *μ*L of ECM. hfPMVECs were mixed 1:1 with trypan blue and viable cells were counted using a hemocytometer.

### Endothelial tube formation/angiogenesis assay

Angiogenesis was assessed using endothelial tube formation assays with hfPMVEC seeded in matrigel. Matrigel growth factor‐reduced (GFR) extracellular matrix (Corning, Product #356231, Lot#: 7009616, Corning, NY) was thawed overnight on ice at 4°C. The following day, 50 *μ*L of Matrigel was added to each well of a pre‐chilled 96‐well cell culture plate. The 96‐well plate was incubated at 37°C for 1 h. In some experiments, ADMA 300* μ*mol/L, 0.1* μ*mol/L DETA NONOate, Z‐DEVD‐FMK 100 *μ*mol/L, and/or vehicle were added to cell culture media overnight and then again before seeding hfPMVECs in matrigel. hfPMVEC were washed with DPBS and re‐suspended in ECM + 1% Fetal Bovine Serum (FBS). Cells (1.5 × 10^4^) were seeded into each Matrigel‐coated well. The plate was incubated at 37°C 21% O_2_‐5% CO_2_‐balance N_2_ at 37°C. After 4–6 h, five pictures of the tubes formed per well were taken using an inverted microscope camera. Quantification was done by counting tube branches (capillary‐like structures) per field of view. The branches were counted using counter plugin in the NIH ImageJ software (ImageJ, 1.47 Version, National Institutes of Health, Bethesda, MD).

### Nitrite assay

Cell media was assayed for nitrite (${\text{NO}}_{2}^{-}$) concentration using a chemiluminescence NO analyzer (Sievers, Boulder, CO), as described previously (Nelin et al. 2007; Jin et al. 2015; Trittmann et al. 2016b). Briefly, 100 *μ*L of sample was placed in a reaction chamber containing a mixture of NaI in glacial acetic acid to reduce nitrite (${\text{NO}}_{2}^{-}$) to NO. The NO gas was carried into the NO analyzer using a constant flow of helium. The analyzer was calibrated using a NaNO_2_ standard curve.

### Statistical analysis

Values are expressed as the means ± SE. One‐way ANOVA was used to compare the data between more than two groups. Significant differences were identified using a Neuman‐Keuls post hoc test. Student's *t*‐test was used to compare means of two groups (SigmaPlot, 12.5 Version, Systat Software, San Jose, CA). Differences were considered significant at *P* < 0.05.

## Results

### DDAH1 knockdown decreased NO production

**Figure 1:**
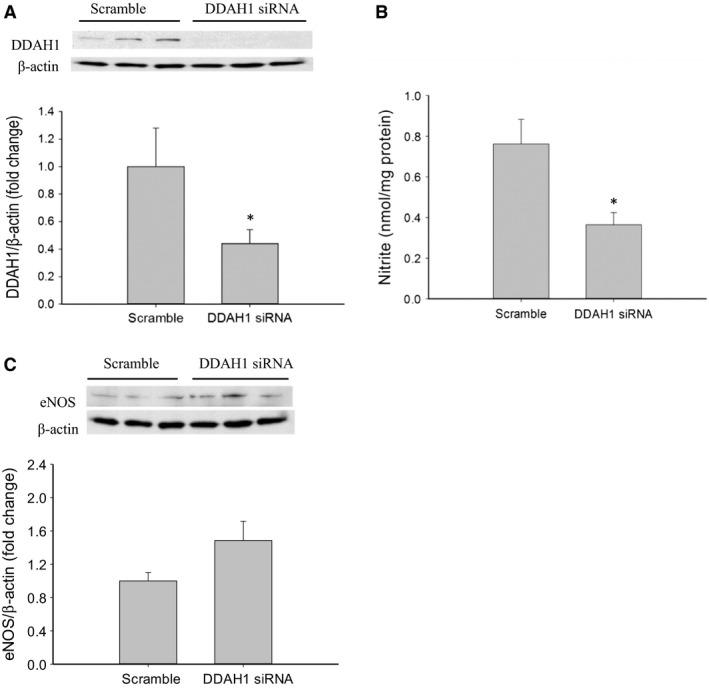
siRNA‐mediated knockdown of DDAH1 in hfPMVEC resulted in less NO production with no significant change in eNOS protein levels. (A) DDAH1 siRNA transfection of hfPMVECs resulted in effective knockdown of DDAH1 protein levels. hfPMVECs were transfected with either scramble siRNA or DDAH1 siRNA for 24 h, recovered for 24 h, and protein isolated. Representative western blot and bar graph of densitometry data for DDAH1 protein levels normalized to *β*‐actin (*n* = 6 in each group). *P < 0.05, DDAH1 siRNA different from scramble. (B) DDAH1 knockdown with siRNA resulted in lower NO production than scramble transfected cells. hfPMVECs were transfected with either scramble siRNA or DDAH1 siRNA for 24 h, washed and allowed to recover for 24 h. Cell were incubated for an additional 24 h and cell media harvested for determination of nitrites using a chemiluminescence NO analyzer. Nitrite levels were normalized to protein concentration in each plate (*n* = 3 in each group). *P < 0.05, DDAH1 siRNA different from scramble. (C) DDAH1 siRNA transfection of hfPMVECs resulted in no significant change of eNOS protein levels. The protein isolated under B above was used in western blotting for eNOS protein levels. Representative western blot and bar graph of densitometry data for eNOS protein level normalized to *β*‐actin (*n* = 6 in each group).

First, to demonstrate knockdown of DDAH1 using siRNA, hfPMVECs were transfected with DDAH1 siRNA or scramble for 24 h. After recovery, hfPMVECs were harvested and protein isolated for western blot analysis of DDAH1 protein levels. As expected, DDAH1 siRNA transfected hfPMVECs had substantially lower protein levels of DDAH1 (Fig. 1A). To determine if DDAH1 knockdown would result in lower NO production, hfPMVECs were transfected with DDAH1 siRNA or scramble. After 24 h, the cells were washed, fresh media placed on them and were incubated for 24 h. Cell media was harvested for determination of the amount of nitrite as a measure of NO production over the 24 h incubation period. Protein was also harvested from the cells for normalization of amount of nitrite in the media to total protein level of each plate. The hfPMVEC transfected with DDAH1 siRNA had significantly lower levels of nitrite in the media than did scramble treated hfPMVEC (Fig. 1B). To assess whether the DDAH1 siRNA had any effects on the levels of eNOS protein in hfPMVEC, some of the protein harvested from the above experiment was used for eNOS western blotting. The hfPMVECs transfected with DDAH1 siRNA had levels of eNOS protein that were not significantly different (*P* = 0.084) from the scramble treated hfPMVEC (Fig. 1C).

### DDAH1 knockdown resulted in lower cleaved caspase‐3 and ‐8 protein levels

**Figure 2:**
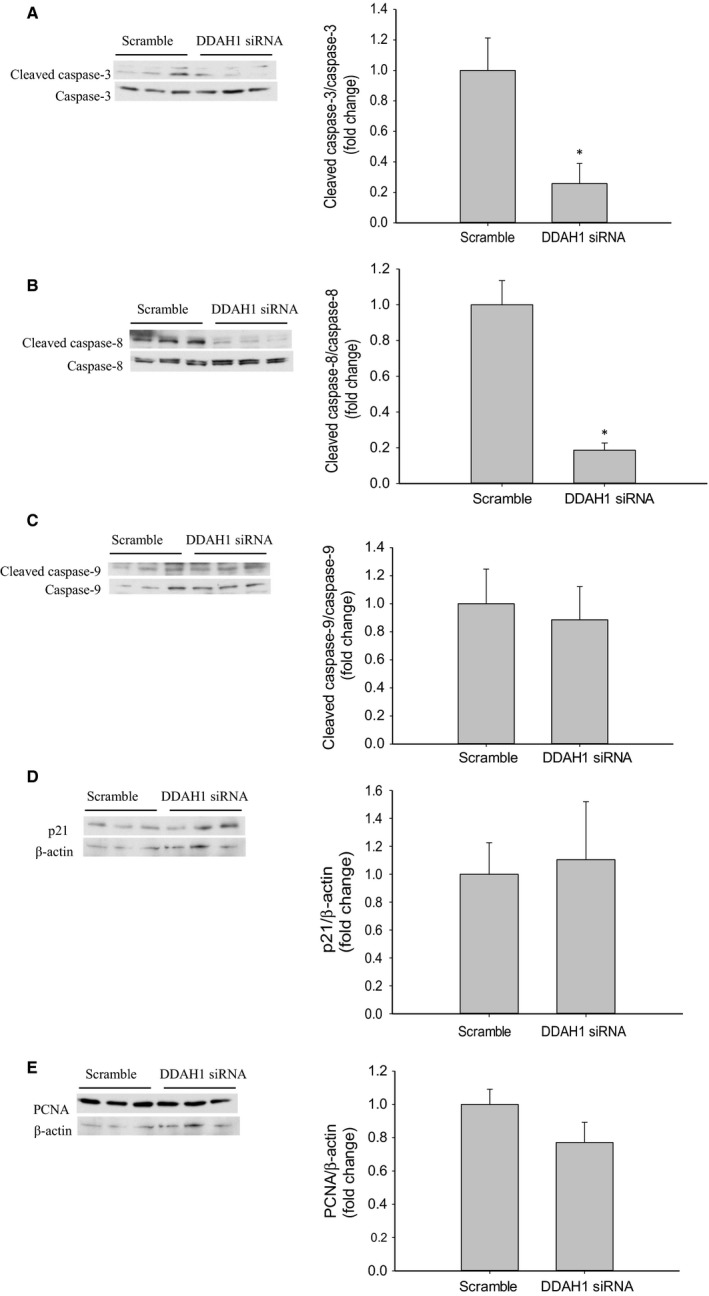
siRNA‐mediated knockdown of DDAH1 in hfPMVEC resulted in lower cleaved caspase‐3 and cleaved caspase‐8 protein levels. hfPMVECs were transfected with either scramble siRNA control or DDAH1 siRNA for 24 h, allowed to recover for 24 h, and then incubated for 24 h and protein isolated for western blot analysis. (A) Transfection with DDAH1 siRNA resulted in lower cleaved caspase‐3 protein levels. Representative western blot and bar graphs for cumulative densitometry data of cleaved caspase‐3 protein levels normalized to total caspase‐3 protein levels (*n* = 6 in each group). *P < 0.05, DDAH1 siRNA different from scramble. (B) Transfection with DDAH1 siRNA resulted in lower cleaved caspase‐8 protein levels. Representative western blot and bar graphs for cumulative densitometry data of cleaved caspase‐8 protein levels normalized to caspase‐8 (*n* = 6 in each group). *P < 0.05, DDAH1 siRNA different from scramble. (C) Transfection with DDAH1 siRNA resulted in no significant change in cleaved caspase‐9 protein levels. Representative western blot and bar graphs for cumulative densitometry data of cleaved caspase‐9 protein levels normalized to caspase‐9 (*n* = 6 in each group). (D) Transfection with DDAH1 siRNA resulted in no significant change in p21 protein levels. Representative western blot and bar graphs for cumulative densitometry data of p21 protein levels normalized to *β*‐actin (*n* = 6 in each group). (E) Transfection with DDAH1 siRNA resulted in no significant change in PCNA protein levels. Representative western blot and bar graphs for cumulative densitometry data of PCNA protein levels normalized to *β*‐actin (*n* = 6 in each group).

To determine the effect of DDAH1 knockdown on hfPMVEC apoptosis, hfPMVECs were transfected with DDAH1 siRNA or scramble for 24 h, allowed to recover for 24 h, and then incubated for 24 h. Protein was then harvested for western blotting. The hfPMVEC transfected with DDAH1 siRNA had significantly lower protein levels of cleaved caspase‐3 than did scramble treated hfPMVEC, while protein levels of total caspase‐3 were similar between groups (Fig. 2A). Similarly, hfPMVEC transfected with DDAH1 siRNA had significantly lower levels of cleaved caspase‐8 protein than did scramble treated hfPMVEC, while protein levels of total caspase‐8 were similar between groups (Fig. 2B). hfPMVEC transfected with DDAH1 siRNA had protein levels of both cleaved caspase‐9 and total caspase‐9 similar to those from hfPMVEC transfected with scramble (Fig. 2C). We also examined p21 and PCNA for their roles in cell proliferation and found that hfPMVECs transfected with DDAH1 siRNA had protein levels of both p21 (Fig. 2D) and PCNA (Fig. 2E) that were not different from scramble transfected hfPMVEC.

### DDAH1 knockdown increased viable cell numbers

**Figure 3:**
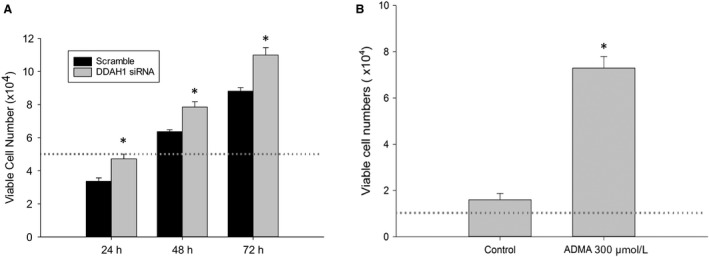
DDAH1 transfection of hfPMVEC resulted in greater viable cell numbers. (A) hfPMVEC transfected with DDAH1 siRNA had greater viable cell numbers at 24, 48, and 72 h than scramble control. hfPMVEC were transfected with DDAH1 siRNA or scramble. After 24 h, the cells were washed, trypsinized, and 5 × 10^4^ cells loaded in each well of a six‐well plate. After 24, 48, or 72 h, viable cell numbers were determined by trypan blue exclusion (*n* = 3 in each group). *P < 0.05, DDAH1 siRNA different from scramble. (B) Non‐transfected hfPMVEC were seeded at 1 × 10^4^ in each well of a six‐well plate and treated with 300 *μ*mol/L ADMA for 24 h or no treatment controls. ADMA‐treated cells had greater viable cell numbers than no treatment controls (*n* = 5 in each group). *P < 0.05, different from control. Dotted line represents seeded cell number.

Although p21 and PCNA levels did not differ between DDAH1 siRNA and scramble hfPMVEC, given the differences in levels of cleaved caspase‐3 protein between DDAH1 siRNA and scramble transfected cells, we hypothesized that viable cells numbers would be greater in DDAH1 siRNA transfected cells than in scramble transfected cells. To test this hypothesis, hfPMVECs were transfected with DDAH1 siRNA or scramble. After 24 h, the cells were washed, trypsinized, and 5 × 10^4^ cells loaded in each well of a six‐well plate. After 24, 48, or 72 h, viable cell numbers were determined by trypan blue exclusion. In both the scramble transfected and DDAH1 siRNA transfected hfPMVECs, there slightly lower numbers of viable cells at 24 h then were plated, thereafter viable cell numbers were significantly greater at each time‐point studied (Fig. 3A). Furthermore, DDAH1 siRNA transfection resulted in significantly more viable cells than in scramble transfected cells at the same time‐point for all time‐points studied (Fig. 3A). In a parallel set of experiments exogenous ADMA or vehicle (control) was added to the cell culture media of non‐transfected hfPMVEC and viable cell numbers determined. The same number of non‐transfected hfPMVECs (for this experiment 1 × 10^4^ cells) were seeded in each well of a six‐well plate and either vehicle or 300* μ*mol/L ADMA were added to the media. After 48 h, the viable cell numbers were determined using trypan blue exclusion. Treatment with ADMA resulted in substantially greater numbers of viable cells than were found in the vehicle treated hfPMVECs (Fig. 3B).

### DDAH1 knockdown decreased angiogenesis

**Figure 4:**
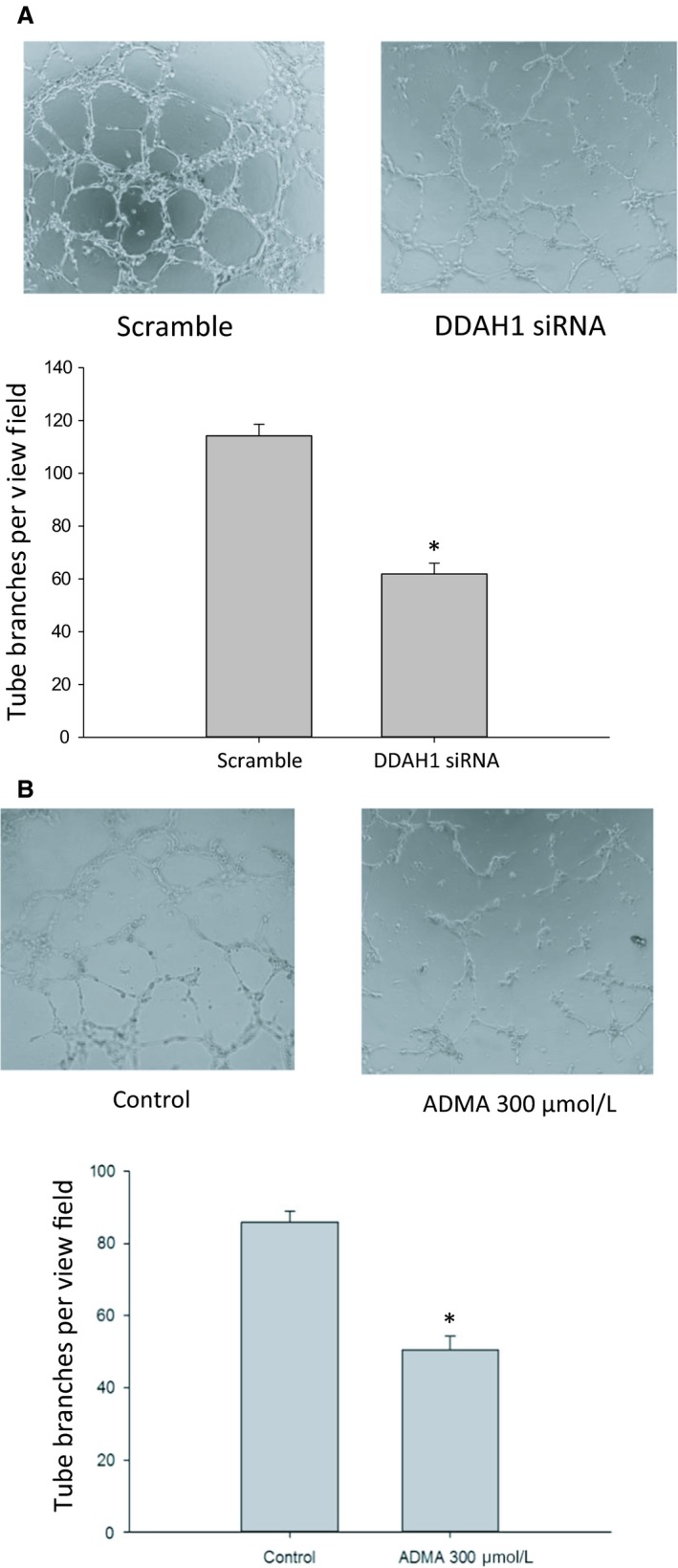
hfPMVEC tube formation was lower in cells transfected with DDAH1 siRNA. (A) hfPMVECs were transfected with either scramble siRNA control or DDAH1 siRNA for 24 h. hfPMVECs were washed with DPBS, trypsinized, and resuspended in ECM + 1% FBS. 1.5 × 10^4^ hfPMVEC were seeded into Matrigel‐coated well on a 96‐well cell culture plate. The plates were incubated at 37°C 21% O_2_‐5% CO_2_‐balance N_2_ for 4–6 h at 37°C. Quantification was performed by counting tube branches per view field (*n* = 6 in each group) using NIH ImageJ software (ImageJ, 1.47 Version, National Institutes of Health, Bethesda, MD). hfPMVEC transfected with DDAH1 siRNA had less endothelial tube formation as measured by tube branches per view field than scramble control. *P < 0.05, DDAH1 siRNA different from scramble. (B) Non‐transfected hfPMVECs were treated with either vehicle or 300 *μ*mol/L ADMA for 24 h and tube branches determined as in A above. hfPMVEC treated with 300* μ*mol/L ADMA had fewer endothelial tubes as measured by tube branches per field of view than did vehicle treated controls. *P < 0.05, different from scramble control.

To determine the effect of siRNA‐mediated DDAH1 knockdown on angiogenesis, hfPMVECs were transfected with DDAH1 siRNA or scramble for 24 h. The cells were then washed, fresh media placed on them, and the cells were allowed to recover for 24 h. After recovery, equal numbers of hfPMVECs (1.5 × 10^4^) were placed in matrigel in each well of a 96‐well plate. After 4 h, tube branches were counted as a measure of angiogenesis. hfPMVEC transfected with DDAH1 siRNA demonstrated some tube formation but not nearly to the extent as seen in cells transfected with scramble (Fig. 4A). The DDAH1 siRNA transfected cells had significantly fewer tube branches than did hfPMVEC transfected with scramble (Fig. 4A). In a second set of experiments, the effect of exogenous ADMA treatment on angiogenesis was studied. Non‐transfected hfPMVECs were treated with either vehicle or 300 *μ*mol/L ADMA for 24 h and tube branches determined as above. After 4 h in matrigel, the hfPMVECs treated with 300* μ*mol/L ADMA had some tube formation but not nearly to the extent of vehicle treated hfPMVEC (Fig. 4B). The ADMA treated cells had significantly fewer tube branches per field of view than did hfPMVEC treated with vehicle (Fig. 4B).

### Treatment with an NO donor restored cleaved caspase 3 levels in DDAH1 siRNA transfected hfPMVEC

**Figure 5:**
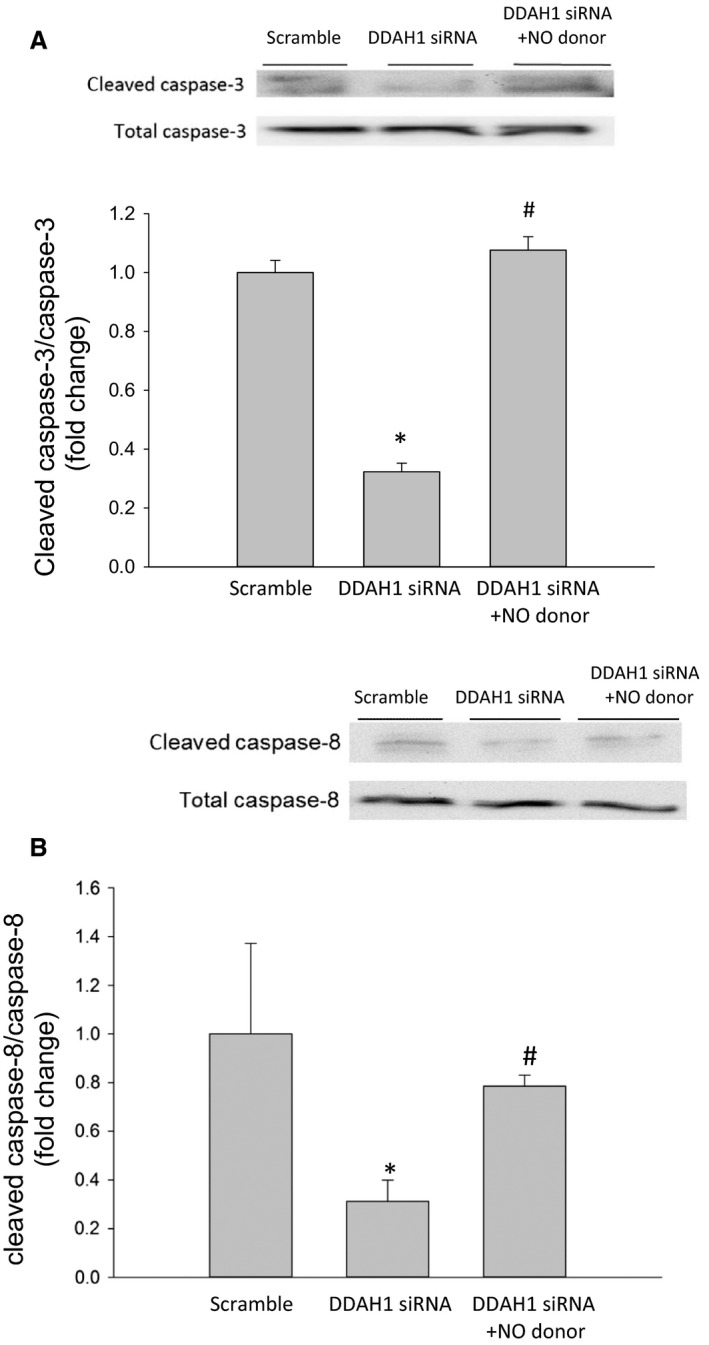
Treatment with an NO donor restored cleaved caspase‐3 and cleaved caspase‐8 protein levels in DDAH1 transfected hfPMVEC. hfPMVEC were transfected with DDAH1 siRNA or scramble for 24 h and then recovered for 24 h. The DDAH1 siRNA transfected hfPMVECs were then treated with either vehicle or 0.1 *μ*mol/L DETA NONOate, while untreated scramble transfected cells were used as a control. After a 24‐h incubation period, protein was isolated and western blotting done for cleaved and total caspase‐3 and ‐8. (A) Addition of the NO donor to DDAH1 siRNA transfected cells resulted in protein levels of cleaved caspase‐3 similar to those seen in the scramble control and significantly greater than DDAH1 siRNA transfected hfPMVEC treated with vehicle. Representative western blot and bar graphs for cumulative densitometry data for cleaved caspase‐3 protein levels normalized to total caspase‐3 (*n* = 3 in each group). *P < 0.05, DDAH1 siRNA different from scramble; ^#^ *P* < 0.05, DDAH1 siRNA + NO donor different from DDAH1 siRNA. (B) Addition of the NO donor to the DDAH1 siRNA transfected hfPMVEC resulted in protein levels of cleaved caspase‐8 similar to those seen in scramble control and significantly greater than DDAH1 siRNA transfected hfPMVEC. Representative western blot and bar graphs for cumulative densitometry data for cleaved caspase‐8 protein levels normalized to caspase‐8 (*n* = 3 in each group). *P < 0.05, DDAH1 siRNA different from scramble; ^#^ *P* < 0.05, DDAH1 siRNA + NO donor different from DDAH1 siRNA.

Given the effects of DDAH1 siRNA transfection on cleaved caspase‐3 protein levels in hfPMVEC, we hypothesized that providing exogenous NO to the DDAH1 siRNA transfected cells would result in greater cleaved caspase‐3 protein levels than in vehicle treated DDAH1 transfected cells. To test this hypothesis DDAH1 siRNA transfected hfPMVECs were treated with the NO donor, DETA NONOate. hfPMVEC were transfected with DDAH1 siRNA or scramble for 24 h and then recovered for 24 h. The DDAH1 siRNA transfected hfPMVECs were then treated with either vehicle or 0.1 *μ*mol/L DETA NONOate, while untreated scramble transfected cells were also studied. After a 24‐h incubation period, protein was isolated and western blotting done for cleaved and total caspase‐3 and ‐8. Once more, hfPMVEC transfected with DDAH1 siRNA had lower levels of cleaved caspase‐3 and ‐8 protein than did scramble transfected cells (Fig. 5A). However, when DDAH1 siRNA transfected hfPMVEC were treated with the NO donor the protein levels of cleaved caspase‐3 were significantly greater than in the DDA1 siRNA transfected vehicle treated cells and were not different from that found in scramble transfected hfPVMEC (Fig. 5A). Similarly, treatment of the DDAH1 siRNA transfected cells with the NO donor resulted in significantly greater protein levels of cleaved caspase‐8 than in DDAH1 transfected cells treated with vehicle, and again the protein levels of cleaved caspase‐8 in the DDAH1 transfected NO donor treated cells were not different from those found in the scramble transfected hfPMVEC (Fig. 5B).

### Treatment with an NO donor decreased viable cell numbers in DDAH1 siRNA transfected hfPMVEC

**Figure 6:**
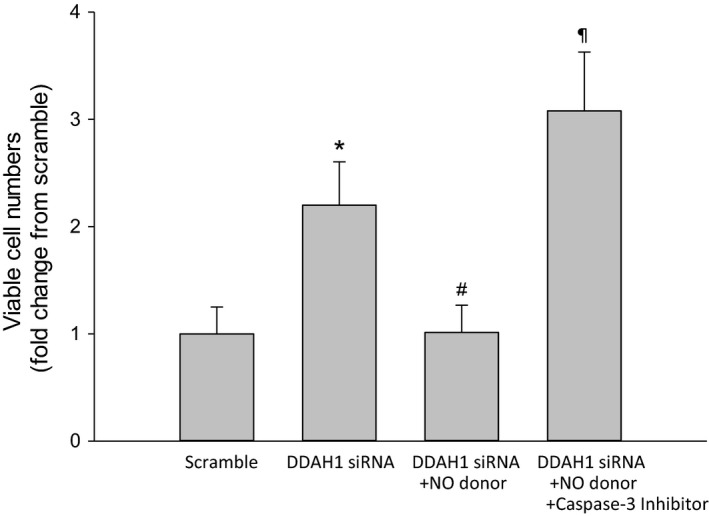
Treatment with an NO donor decreased viable cell numbers in DDAH1 siRNA transfected cells. hfPMVEC were transfected with either DDAH1 siRNA or scramble for 24 h, the cells were washed and allowed to recover for 24 h. The cells were then trypsinized and equal numbers seeded in each well of a six‐well plate. Some DDAH1 siRNA transfected cells were treated with 0.1 *μ*mol/L DETA NONOate and some were treated with both 0.1 *μ*mol/L DETA NONOate and 100 *μ*mol/L Z‐DEVD‐FMK. After 72 h, viable cell numbers were counted using trypan blue exclusion (*n* = 6, each group) and presented as fold change compared to scramble. Addition of the NO donor to hfPMVEC transfected with DDAH1 siRNA resulted in significantly lower viable cell numbers than vehicle treated DDAH1 siRNA‐transfected hfPMVEC. Addition of the caspase‐3 inhibitor to NO donor treated DDAH1 transfected hfPMVEC resulted in three‐fold greater viable cell numbers than in either scramble control or the DDAH1 siRNA transfected NO donor‐treated hfPMVEC. *P < 0.05, DDAH1 siRNA different than scramble; ^#^ *P* < 0.05, DDAH1 siRNA + NO donor different than DDAH1 siRNA; ^¶^DDAH1 siRNA + NO donor + caspase‐3 inhibitor different from all other conditions, *P* < 0.05.

Given the effect of the DDAH1 siRNA transfection on viable cell numbers and the effect of the NO donor on cleaved caspase‐3 protein levels in DDAH1 siRNA transfected hfPMVEC, we hypothesized that treatment with an NO donor would decrease viable cell numbers in DDAH1 siRNA transfected cells and that the effect of the NO donor was through its effects on caspase‐3 activity. To test this hypothesis in DDAH1 siRNA transfected hfPMVEC, we again utilized the NO donor, DETA NONOate, as well as the putative caspase‐3 inhibitor Z‐DEVD‐FMK (R&D Systems, Minneapolis, MN). hfPMVEC were transfected with either DDAH1 siRNA or scramble for 24 h, the cells were washed and allowed to recover for 24 h. The cells were then trypsinized and equal numbers seeded in each well of a 6‐well plate. Some DDAH1 siRNA transfected cells were treated with 0.1 *μ*mol/L DETA NONOate and some were treated with both 0.1 *μ*mol/L DETA NONOate and 100 *μ*mol/L Z‐DEVD‐FMK. After 72 h, viable cell numbers were counted using trypan blue exclusion. We again found that DDAH1 siRNA transfected hfPMVEC had substantially greater viable cell numbers than did scramble hfPMVEC (Fig. 6). Addition of the NO donor to the DDAH1 siRNA transfected hfPMVEC resulted in significantly fewer viable cells than in the vehicle treated DDAH1 siRNA transfected cells, and the viable cell number were not different from those seen in the scramble transfected hfPMVEC (Fig. 6). Furthermore, in hfPMVEC transfected with DDAH1 siRNA and treated with both DETA NONOate and Z‐DEVD‐FMK the viable cell numbers were substantially greater than in the DDAH1 siRNA transfected, NO donor treated hfPMVEC (Fig. 6).

### Treatment with an NO donor enhanced tube formation in DDAH1 siRNA transfected hfPMVEC

**Figure 7:**
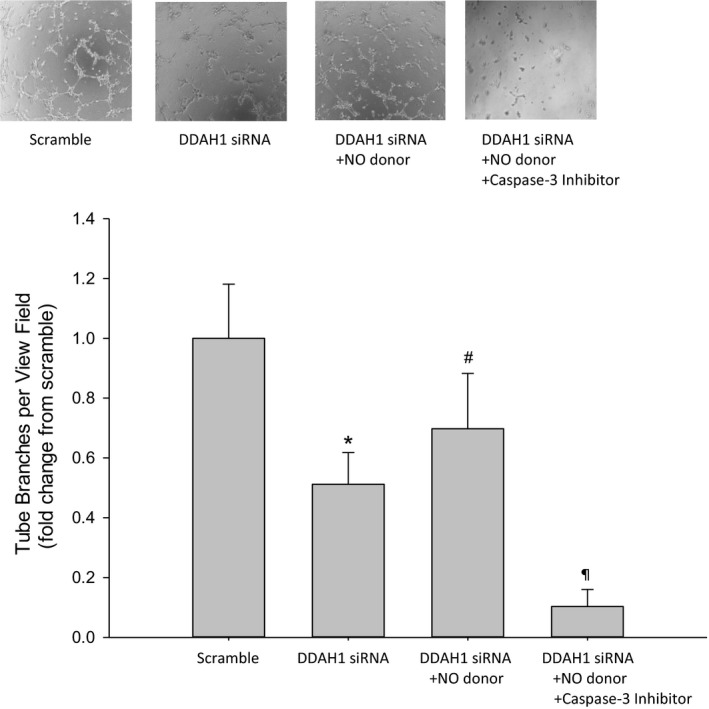
Treatment with the NO donor increased tube branches in DDAH1 siRNA transfected hfPMVEC. hfPMVEC were transfected with either DDAH1 siRNA or scramble for 24 h, and the cells were washed and allowed to recover for 24 h. hfPMVEC were then trypsinized and equal numbers seeded in matrigel in each well of a 96‐well plate. Some of the DDAH1 siRNA transfected cells had 0.1 *μ*mol/L DETA NONOate added and some of the DDAH1 transfected cells had both 0.1 *μ*mol/L DETA NONOate and 100 *μ*mol/L Z‐DEVD‐FMK added. Treatments were given to cell media both prior to putting the cells in matrigel and again during the incubation in matrigel. After 4 h, the number of branches were determined. Quantification was performed by counting tube branches per view field (*n* = 6 in each group) and presented as fold change compared to scramble. hfPMVEC transfected with DDAH1 siRNA and treated with the NO donor had more tube branches per field of view than did vehicle treated DDAH1 siRNA‐transfected hfPMVEC. hfPMVEC transfected with DDAH1 siRNA and treated with both the NO donor and the caspase‐3 inhibitor had fewer tube branches per field of view than any of the other groups. *P < 0.05, DDAH1 siRNA different from scramble; ^#^ *P* < 0.05, DDAH1 siRNA + NO donor different from DDAH1 siRNA; ^¶^DDAH1 siRNA + NO donor + caspase‐3 inhibitor different from all other conditions, *P* < 0.05.

Given the effect of DDAH1 siRNA on tube formation, we hypothesized that treating DDAH1 siRNA transfected cells with an NO donor would result in greater tube formation and that this effect of the NO donor would be through caspase‐3 activation. To test this hypothesis, we again utilized the NO donor, DETA NONOate, and the putative caspase‐3 inhibitor, Z‐DEVD‐FMK. The hfPMVEC were transfected with either DDAH1 siRNA or scramble for 24 h, and the cells were washed and allowed to recover for 24 h. The hfPMVEC were then trypsinized and equal numbers seeded in matrigel in each well of a 96‐well plate. Some of the DDAH1 siRNA transfected cells had 0.1 *μ*mol/L DETA NONOate added and some of the DDAH1 transfected cells had both 0.1 *μ*mol/L DETA NONOate and 100 *μ*mol/L Z‐DEVD‐FMK added. Treatments were given to cell media both prior to putting the cells in matrigel and again during the incubation in matrigel. After 4 h, the number of branches were determined. Again, DDAH1 siRNA treated cells had significantly fewer branches than did scramble transfected cells (Fig. 7). hfPMVEC transfected with DDAH1 siRNA and treated with the NO donor had significantly more branches than did hfPMVEC transfected with DDAH1 siRNA (Fig. 7). hfPMVEC transfected with DDAH1 siRNA and treated with both the NO donor and the caspase‐3 inhibitor had very few branches per field of view and substantially fewer than the DDAH1 siRNA transfected hfPMVEC with DETA NONOate added to the media (Fig. 7).

## Discussion

**Figure 8:**
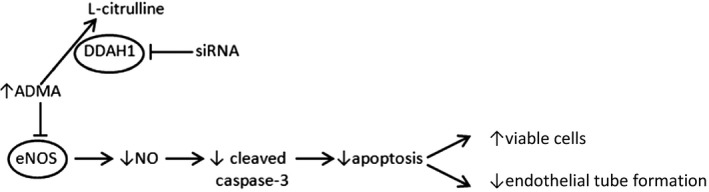
Proposed model of DDAH1 inhibition of the NO‐mediated activation of cleaved caspase‐3 and resultant effects on viable cell numbers and angiogenesis. DDAH1 inhibition leads to an accumulation of ADMA, which inhibits eNOS function to produce less NO, this leads to lower levels of activated caspase‐3. Less activated caspase‐3 leads to more viable cells and decreased endothelial tube formation.

The main findings of this study were that knockdown of DDAH1 and/or treatment with exogenous ADMA in hfPMVEC: (1) decreased nitrite levels without significantly effecting eNOS expression; (2) decreased protein levels of cleaved caspase‐3 and ‐8; (3) resulted in greater numbers of viable hfPMVEC; and (4) decreased hfPMVEC angiogenesis. Furthermore, we found that: (1) an NO donor restored cleaved caspase‐3 and 8 levels, attenuated viable hfPMVEC number, and enhanced hfPMVEC angiogenesis following DDAH1 knockdown and (2) a caspase‐3 inhibitor enhanced hfPMVEC viable cell number and attenuated hfPMVEC angiogenesis after DDAH1 knockdown and NO donor treatment. We found that DDAH1 knockdown resulted in lower levels of NO production without significant effects on eNOS protein levels. These data are consistent with our hypothesis that knockdown of DDAH1 in hfPMVEC led to accumulation of ADMA and resultant inhibition of eNOS activity. Taken together our data support a model of DDAH1 regulation of apoptosis and angiogenesis through NO‐mediated caspase‐3 activation in hfPMVEC (Fig. 8).

We found that DDAH1 knockdown attenuated the activation of caspase‐3 and ‐8, which was associated with an increase in viable cell numbers. Caspase‐3 is the effector of apoptotic cell death, and caspase‐8 is activated by a death receptor through the extrinsic apoptotic pathway, which in turn activates cleaved caspase‐3 (Cullen and Martin 2009). Given that we found essentially no changes in activation of caspase‐9, this suggests that the NO‐mediated activation of caspase‐3 occurs through the extrinsic apoptotic pathway in non‐stimulated hfPMVEC. Targeted therapies to enhance apoptosis in the vascular wall and thereby improve angiogenesis may have therapeutic potential in BPD‐PH. Although one such therapy, inhaled NO (iNO), which would likely act similar to the NO donor in these experiments to enhance apoptosis, has not been found to decrease the incidence of BPD per se (Hasan et al. 2017). However, iNO has not been studied specifically for the prevention of BPD‐PH. Previous studies have suggested that untreated hPMVECs from children and adults have low levels of cleaved caspase‐3 protein expression, however after treatment with LPS, IL‐1*β*, TNF‐*α*, and IFN‐*γ*, cleaved caspase‐3 was substantially induced (Nelin et al. 2016). In the present study, we found that unstimulated hfPMVEC had basal activation of caspase‐3 as evidenced by readily detectable protein levels of cleaved caspase‐3. Thus, it may be that fetal PMVEC differ from adult PMVEC in the levels of caspase‐3 activation. In a study on fetal rat lungs, activated caspase‐3 was expressed from E15 corresponding roughly to the pseudoglandular period to the day of birth E21 (Stiles et al. 2001), as were proteins related to proliferation. This led the authors to speculate that proliferation is the primary driving process during fetal lung development with apoptosis occurring throughout to refine structural remodeling (Mourani and Abman 2015). Thus, it seems that normal fetal lung development requires a balance between proliferation and apoptosis, and in hfPMVEC this balance is regulated, at least in part, by DDAH1 effects on NO production. While mice with an endothelial cell specific DDAH1 knock out had normal lung development (Hu et al. 2009), we speculate that in the face of a stimulus in the neonatal lung (i.e., hyperoxic exposure), endothelial specific DDAH1 knockout mice may be more prone to develop BPD and BPD‐PH by attenuation of apoptosis. This postulate is consistent with our findings in patients with BPD that a mutation in DDAH1 affects the incidence of BPD‐PH (Trittmann et al. 2016a).

We found that DDAH1 knockdown in hfPMVEC resulted in less angiogenesis, which was restored by treatment with an NO donor, and greatly reduced following treatment with a caspase‐3 inhibitor, again supporting a role for NO enhancing hfPMVEC angiogenesis via activation of caspase‐3. In a study using bovine aortic endothelial cells, the NOS inhibitor L‐NAME prevents endothelial cell sprouting as measured by the scratch test, and inhibited endothelial cell migration as measured by Boyden chamber experiments (Murohara et al. 1999). In studies using tumor cell lines after irradiation, caspase‐3 positively regulated VEGF to promote angiogenesis (Feng et al. 2015, 2017). These findings are consistent with our results that DDAH1 siRNA led to decreased NO production and decreased caspase‐3 activation in hfPMVEC, which resulted in an attenuation of angiogenesis through DDAH1‐mediated decreased NO‐induced activation of caspase‐3. Angiogenesis is a multi‐step process that includes sprouting of new endothelium, as well as intussusceptive microvascular growth characterized by dividing existing vessel lumens. Angiogenesis is the primary mechanism for pulmonary lung growth in the second half of gestation. Alterations in pulmonary vascular development are a major contributor to BPD‐PH and apoptosis is known to be important in blood vessel formation and maintenance (Teng et al. 2009). Premature infants are particularly susceptible to pulmonary vascular injury since they are born during the saccular stage, or in extremely preterm infants the canalicular stage, of lung development when a peak number of new lung capillaries are in the process of being formed (Goss 2018). Angiogenesis over time produces the generation of new pulmonary vasculature that supports alveolar development (Thebaud 2007). Consistent with our findings in hfPMVEC, studies in pulmonary artery endothelial cells isolated from fetal lambs demonstrated that when NO production was decreased angiogenesis was reduced (Teng et al. 2011b) and when NO production was augmented angiogenesis was increased (Teng et al. 2011a). Taken together these results demonstrate that NO production is involved in angiogenesis in hfPMVEC.

In summary, we describe an important regulatory role for DDAH1 in hfPMVEC through regulation of NO production which in turn regulates caspase‐3 activation, apoptosis, and angiogenesis. We found by decreasing DDAH1 expression levels using siRNA and thereby putatively decreasing DDAH1 activity, that NO production was decreased resulting in less caspase‐3‐induced apoptosis and less angiogenesis. We found that the NO‐mediated caspase‐3 activation occurred via the extrinsic apoptotic pathway in non‐stimulated hfPMVEC. Of course, further study is needed, but our findings suggest that DDAH1 potentially could be a viable therapeutic target for preventative and/or therapeutic treatments for lung diseases of the preterm infant.

## Conflict of Interest

No conflicts of interest are declared by the authors.
